# Behavioural Effects of Tourism on Oceanic Common Dolphins, *Delphinus* sp., in New Zealand: The Effects of Markov Analysis Variations and Current Tour Operator Compliance with Regulations

**DOI:** 10.1371/journal.pone.0116962

**Published:** 2015-01-07

**Authors:** Anna M. Meissner, Fredrik Christiansen, Emmanuelle Martinez, Matthew D. M. Pawley, Mark B. Orams, Karen A. Stockin

**Affiliations:** 1 Coastal-Marine Research Group, Institute of Natural and Mathematical Sciences, Massey University, Private Bag 102904, North Shore City, Auckland, New Zealand; 2 Centre for Integrative Ecology, School of Life and Environmental Sciences, Deakin University, Warrnambool Campus, PO Box 423, Warrnambool, Victoria, Australia; 3 Cetacean Research Unit, School of Veterinary and Life Sciences, Murdoch University, South Street, Western Australia, Australia; 4 Pacific Whale Foundation, 300 Ma’alaea Rd., Suite 211, Wailuku, Hawaii, United States of America; 5 New Zealand Tourism Research Institute, School of Hospitality and Tourism, Auckland University of Technology, Private Bag 92006, Auckland, New Zealand; Institut National de la Recherche Agronomique, FRANCE

## Abstract

Common dolphins, *Delphinus* sp., are one of the marine mammal species tourism operations in New Zealand focus on. While effects of cetacean-watching activities have previously been examined in coastal regions in New Zealand, this study is the first to investigate effects of commercial tourism and recreational vessels on common dolphins in an open oceanic habitat. Observations from both an independent research vessel and aboard commercial tour vessels operating off the central and east coast Bay of Plenty, North Island, New Zealand were used to assess dolphin behaviour and record the level of compliance by permitted commercial tour operators and private recreational vessels with New Zealand regulations. Dolphin behaviour was assessed using two different approaches to Markov chain analysis in order to examine variation of responses of dolphins to vessels. Results showed that, regardless of the variance in Markov methods, dolphin foraging behaviour was significantly altered by boat interactions. Dolphins spent less time foraging during interactions and took significantly longer to return to foraging once disrupted by vessel presence. This research raises concerns about the potential disruption to feeding, a biologically critical behaviour. This may be particularly important in an open oceanic habitat, where prey resources are typically widely dispersed and unpredictable in abundance. Furthermore, because tourism in this region focuses on common dolphins transiting between adjacent coastal locations, the potential for cumulative effects could exacerbate the local effects demonstrated in this study. While the overall level of compliance by commercial operators was relatively high, non-compliance to the regulations was observed with time restriction, number or speed of vessels interacting with dolphins not being respected. Additionally, prohibited swimming with calves did occur. The effects shown in this study should be carefully considered within conservation management plans, in order to reduce the risk of detrimental effects on common dolphins within the region.

## Introduction

Over the past two decades, an abundance of literature referring to boat based marine mammal tourism has clearly shown that cetacean-watching is seldom benign and that careful management is required to minimise potential negative effects on targeted populations [1, 2, 3, 4, 5, 6, 7]. Vessel presence has for example been shown to increase dolphin travelling behaviour at the expenses of foraging [8, 9], resting [9, 10] or socialising [11, 12]. Authors have also reported some species avoiding approaching vessels [8, 13, 14]. Although the risk of ship strikes has long been a concern for larger whales [15], collisions between small delphinids and tour vessels [16] or recreational craft [17] have also been reported. Tourism also exposes cetaceans to noise pollution which may lead to chronic auditory damage [18, 19] or to exhaust emissions that are likely to cause serious health effects [20]. Close encounters with wild cetaceans at sea have also become more and more intrusive, including swimming [21, 22, 23] or provisioning dolphins with food, whether monitored or illegal [24, 25], leading to possibly dangerous situations for both dolphins and humans [24, 26, 27, 28, 29, 30, 31]. Although viewing and swimming activities are regarded as relatively safe from an infectious standpoint [32], serious concerns have been raised as increased opportunities for disease transmissions exist and dolphins could potentially be infected by humans [33].

Recent tourism impact studies have argued that short-term behavioural changes can have long-term implications for targeted populations by disrupting energy budgets, reducing energy uptake and/or increasing physical demands [34, 35, 36, 37, 38]. As such, there is increasing evidence that individual behavioural changes can potentially lead to population-level effects [3]. However, despite numerous concerns raised by the scientific community, the cetacean-watching industry is still experiencing a fast world-wide expansion, as the economic benefits of marine mammal based-activities represent a significant part of the ecotourism industry [39, 40]. The dolphin-watching industry in Oceania has followed this global trend and is now widespread in 17 countries within this region. In New Zealand alone, approximately 550,000 international and domestic cetacean-watching tourists resulted in over US$80 million in expenditure in 2008 [39]. Permits to watch and/or swim-with-dolphins in New Zealand increased from 90 in 2005 [41] to 112 in 2011 (Young, pers.comm.).

Most marine mammals in New Zealand are the focus of tourism operations, including endemic species such as the Hector’s dolphin, *Cephalorhynchus hectori hectori* [23] and the New Zealand sea lion, *Phocarctos hookeri* [42]. Nationally endangered, the bottlenose dolphin, *Tursiops truncatus*, is also targeted by tourism activities [10, 43, 44]. While the vast majority of scientific studies have evaluated the effects of tourism activities on the behaviour of coastal species [10, 11, 12, 14, 45, 46], since they are believed to be subject to and impacted by human activities to a greater degree than oceanic species, considerable less is known about the effects of tourism activities on pelagic oceanic populations of delphinids [8, 47, 48].

**Figure 1:**
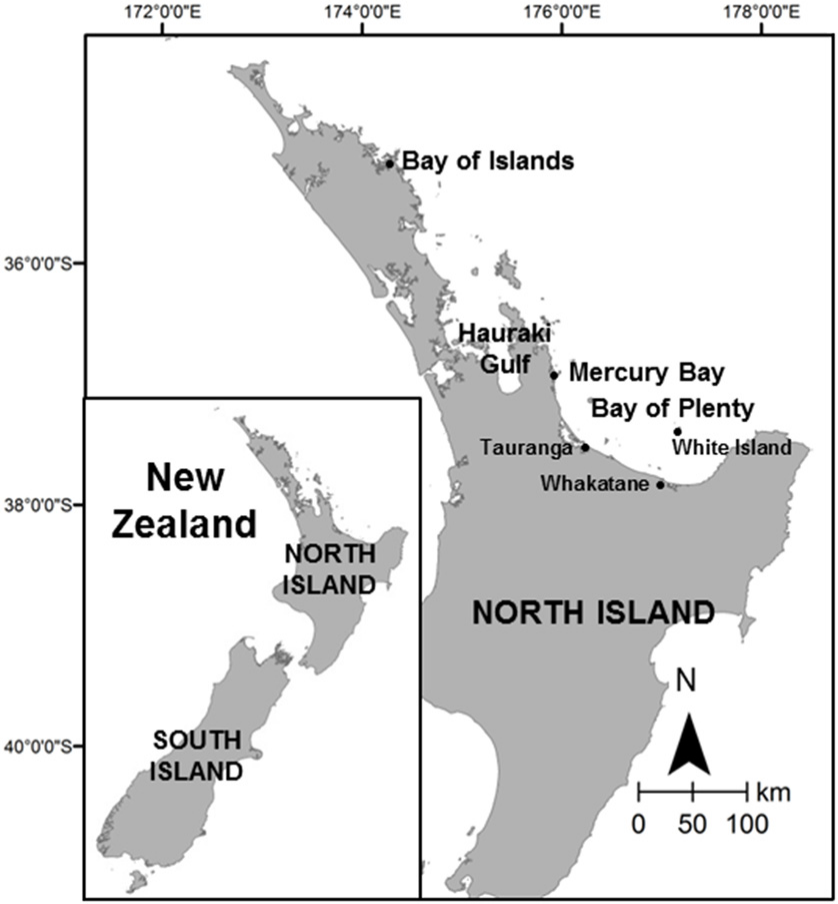
Study area. Location of the Bay of Plenty (BOP) and other places referred to in the text in relation to the North and South Island of New Zealand.

Short- and long-beaked common dolphins, *Delphinus delphis* and *D. capensis*, are listed by the International Union for Conservation of Nature (IUCN) as ‘least concern’ and ‘data deficient’, respectively [49, 50]. Under the New Zealand Threat Classification System [51], common dolphins, *Delphinus* sp., are currently classified as ‘not threatened’[52], despite the absence of density and population estimates [53]. Moreover, they remain the only resident cetacean species within New Zealand to lack a species-specific Marine Mammal Action Plan [54]. Recently, the IUCN classified the Mediterranean common dolphins as ‘endangered’, after the population in the eastern Ionian Sea was discovered to be in decline [55, 56]. Although generally considered to be a pelagic species associated with deep waters [57], common dolphins in many parts of New Zealand use nearshore waters and may therefore be vulnerable to coastal anthropogenic activities such as pollution, fishery by-catch and vessel collision [58, 59, 60]. The effects of tourism activities on common dolphins have previously been examined in northern coastal regions of New Zealand, including the Bay of Islands, the Hauraki Gulf and Mercury Bay [8, 9, 61]. However, with a typical oceanic distribution off the Bay of Plenty (BOP, Fig. 1), common dolphins have been considered as less vulnerable to tourism effects given their offshore movements [62]. Despite their oceanic distribution, common dolphins are the focus of marine mammal tourism operations in the BOP, especially in austral summer when the peak of tourism activities coincides with the species breeding season [63, 64]. Since 1995, eight permits (all commercial marine mammal tour operators require a permit in New Zealand) had been issued by the New Zealand Department of Conservation (DOC).

In the present study, we assessed the level of vessel traffic and interactions in the BOP, including commercial tourism and recreational viewing and swimming activities. We then investigated their effects on the behaviour of common dolphins using open oceanic waters off the BOP to determine if they were less pronounced than those previously demonstrated for this species using inshore coastal waters [8, 9]. For this, we examined variations in the dolphin responses to vessel interaction by applying two approaches of Markov chain analysis. Finally, we assessed compliance of dolphin-viewing and swimming operations in regards to permit conditions and to the New Zealand Marine Mammal Protection Regulations (MMRP) [65], by assessing the number and speed of vessels interacting with a single dolphin group, the duration of the encounters, as well as the occurrence of immature animals during swim trips.

## Research Design and Methods

### Study Site

The BOP, situated in the North Island, New Zealand (Fig. 1), is an important habitat for common dolphins [66] with water depths reaching 250m within 30km off the coastline. The area is an open bay influenced by the East Auckland Current, which follows the coastline south-eastward and transports relatively warm and saline subtropical water [67, 68, 69]. Common dolphins frequent the area throughout the year, but especially during the austral summer [66, 70].

Marine traffic in the BOP consists of a wide variety of vessels. As one of New Zealand’s fastest growing cities and being the largest port in the country in terms of total cargo volume, Tauranga accommodates large commercial ships, fishing boats, ferries, cruise liners, recreational power boats, yachts and other non-motorised craft. Tauranga is also the departure port for seven commercial dolphin tour vessels from November to April essentially, while the coastal township of Whakatane (90km to the south east) is the base for three further commercial vessels, two of which undertake opportunistic dolphin-viewing year round during sight-seeing trips to the active volcano White Island (Fig. 1).

### Data Collection

**Observation platforms.** Non-systematic surveys were conducted between November 2010 and May 2013 from two types of platforms; (i) a research vessel (RV); a 5.5m Stabicraft trailer-launched vessel powered by a 90hp four-stroke engine and (ii) seven commercial tour vessels (TV); four motorised dock-based vessels of 12 to 22.3m, a 15m motorised dock-based catamaran, a 10.5m motorised trailer-launched vessel and an 18m motorised dock-based sailing vessel. The RV operated from Tauranga harbour while the TV ran concurrently from Tauranga harbour (n = 4) and from Whakatane (n = 3). All the field work from aboard the RV was permitted by DOC and conducted in full compliance with the DOC guidelines and New Zealand MMPR [65]. Additional data were collected from aboard TV operating under a DOC permit for the BOP region.

**Focal group follows.** The effects of vessel interactions on dolphin behaviour were only examined from aboard the RV, using focal group scan sampling [71, 72]. Focal individual follows [72] were neither feasible nor appropriate for this study owing to the difficulties of identifying individual common dolphins in the field and the increased probability of disturbing the group when attempting to track one individual [63, 73]. Instead, focal group scan sampling followed established protocols for collecting behavioural data on this species, with scans undertaken with naked eyes from the left to the right in order to include all individuals within the group [73] and to avoid attention being drawn only to conspicuous individuals and/or behaviours [72]. If fission of the focal group occurred, the largest subgroup became the focal group.

A group of dolphins was defined as any number of dolphins observed in association, moving in a similar direction and usually engaged in a similar behaviour [74]. Members were assumed to be part of a group when they remained within 100m of each other [75]. Group size was recorded in the field as a best estimate. Group composition was categorized as adults and immatures (i.e. neonates, calves and/or juveniles), following Stockin *et al*. [76].

Once a focal group follow started, the behavioural state of the dolphin group was assessed using categories modelled on Neumann [63] and Stockin *et al*. [9] (Table 1) and recorded every 3min. The predominant behaviour was determined as the behavioural state in which more than 50.0% of the dolphins within the group were involved at the time of sampling [9, 10]. Where groups exhibited an equal percentage of individuals engaged in different behaviours, all represented behavioural states were recorded. Only behaviours that could be reliably and consistently recorded were sampled [72].

**Table 1:** Definitions of behavioural states of common dolphin groups in the Bay of Plenty, New Zealand modelled on Neumannn [63] and Stockin *et al*. [9].

**Behavioural state**	**Definition**
Foraging	Dolphins involved in any effort to pursue, capture and/or consume prey, as defined by observations of fish chasing (herding), co-ordinated deep and/or long diving and rapid circle swimming. Prey can often be observed at the surface during foraging. High number of non-coordinated re-entry leaps, rapid changes in direction and long dives are observed.
Milling	Dolphins exhibit non-directional movement, frequent changes in bearing prevent animals from making headway in any specific direction. Different individuals within a group can swim in different directions at a given time, but their frequent directional changes keep them together.
Resting	Dolphins observed in a tight group (less than one body length apart), engaged in slow manoeuvres (slower than the idle speed of the observing boat) with little evidence of forward propulsion. Surfacings appear slow and are generally more predictable (often synchronous) than those observed in other behavioural states.
Socialising	Dolphins observed in diverse interactive events among members of the group such as social rub, aggressiveness, chasing, mating and/or engaging in any other physical contact with other dolphins (excluding mother-calf pairs). Aerial behavioural events such as breaching are frequently observed.
Travelling	Dolphins engaged in persistent, directional movement making noticeable headway along a specific compass bearing at a constant speed (usually faster than the idle speed of the observing boat). Group spacing varies and individuals swim with short, relatively constant dive intervals.

A focal group follow constituted one or several sequences, i.e. succession of behavioural states, considered as control sequences in the presence of the RV only, or as interaction sequences when other vessel(s) were present within 300m of the focal group of dolphins [9, 10] while viewing and/or swimming with the dolphins. This distance is consistent with the New Zealand MMPR [65].

All vessels interacting with the dolphins were recorded and categorized as: a) *commercial TV*; b) *non-motorised craft—*kayaks, stand up paddleboards, rowing craft, etc; c) *motorised recreational launches—*inboard vessels; d) *motorised recreational trailer-launched vessels—*outboard vessels less than 8m; e) *motorised personal water craft—*jet skis; f) *motorised commercial vessels—*container ships, commercial fishing vessels, etc. Approximate speed of interacting vessels was estimated in relation to the speed of the RV.

In compliance with the DOC guidelines, the New Zealand MMPR [65] and to minimise effects on dolphin behaviour, consistent and careful handling of the RV was necessary when approaching and following dolphin groups [9, 10]. All focal follows terminated when fuel reserves became low, weather or daylight deteriorated, or when visual contact with the dolphins was lost. The end of an encounter was therefore not dependent on the behaviour of the focal group [9]. This protocol was maintained during vessel interactions and thus the state of the observing RV remained consistent throughout all control and interaction scenarios. Consequently, differences observed in the behaviour of the dolphins were assumed to be related only to the presence of the other interacting vessel(s).

**Swimming with the dolphins.** Under their permits, commercial tour operators performed swim encounters with the dolphins, which consisted of one or several swim attempts. Swim-with-dolphins activities in the BOP are active and boat-based [33, 77]. Tour vessels typically approach parallel or behind the group of dolphins, while assessing for the presence of calves, group behaviour and weather conditions for swimmer safety. Once a decision is made by the skipper to proceed to a swim, swimmers are actively placed in the water, generally holding onto ropes or bars at the stern of the vessel and only occasionally free swimming/snorkelling [70]. The duration of the swim attempt, recorded from the RV and from aboard the TV, commenced when the first swimmer entered the water and ended when the last swimmer got back aboard the boat. Dolphin responses to swimmers were recorded from aboard the TV and adapted from Constantine [21] and Martinez *et al*. [23] as follows: a) *neutral presence—*no apparent change in dolphin behaviour. At least one dolphin remained within 5m of the swimmers for at least 5s. Interaction time was recorded when at least one dolphin was within 5m of the swimmers; b) *neutral absence—*no apparent change in dolphin behaviour. Dolphins were initially more than 5m distant from the swimmers and did not approach within 5m; c) *avoidance—*change in dolphin behaviour. Dolphins were within 5m of the boat and departed as swimmers entered the water; d) *interaction—*change in dolphin behaviour. Dolphins were greater than 5m distant from the swimmers and at least one dolphin approached the swimmers at least once and for at least 5s.

The different reasons for ending a swim encounter were recorded from aboard the TV and categorized as follows: a) *unsuccessful swim due to dolphin behaviour*—fast travelling dolphins could not be pursued, or non-interactive dolphins could not be seen by the swimmers; b) *loss of sight of dolphins*—the dolphin group could not be viewed from the surface; c) *skipper decision*—due to time restrictions, i.e. the maximum time allowed for dolphin encounters was reached, or because swimmers were cold and/or tired; d) *presence of calf(ves)* detected during the swim attempt.

### Regulations applying to commercial tour vessels in the BOP

Under their permit conditions, commercial operators in the BOP are restricted to operate outside Tauranga harbour and interact with dolphins for a maximum of 90min per trip, of which 60min can be used to swim with the dolphins assuming no calves are present in the group. In addition, under the New Zealand MMPR [65], all commercial and recreational vessels are limited to a “non-wake” speed (approximately 5kts) while within 300m of the dolphins and cannot approach the group if three vessels are already engaged with the group (i.e. viewing and/or swimming within 300m of the dolphins).

### Statistical analysis

**Effect of boat interactions.** Markov chain analyses have been widely applied as a technique to explore the potential effects of tourism activities on marine mammals (e.g. [9, 10, 12, 35, 46, 78, 79]). These analyses compare the behaviour of the dolphins both when in the presence and in the absence of tour vessels while simultaneously taking into account the temporal dependence between behavioural states. This is achieved by calculating probabilities of transitions from preceding to succeeding behavioural states [10]. However, as the effect of the approach and departure of vessels on dolphin behaviour remains unclear, authors have considered those specific transitions (going from no boats present to boats present and *vice versa*) differently across the various published studies. A conservative approach eliminates any transition in which the animal state might potentially be uncertain as to whether it is a control or interaction situation (behavioural states following or affected by the approach/departure of a vessel are discarded from the analysis, Table 2) and focuses on examining the transitions in the presence and absence of interacting vessels, respectively [9, 46, 48]. Conversely, other authors consider also the transition in behavioural state at the onset of an interaction (going from no boats present to boats present) as affected [10, 12] (Table 2). In the present study, we examined the effects of vessel interactions using both types of approaches to examine the level of difference in dolphin responses. As Markov chain analysis does not account for multiple behavioural states when collected simultaneously (i.e. when the group was split equally between two behavioural states), double states were excluded from the analyses. The program UNCERT (http://www.animalbehavior.org/Resources/CSASAB/) was used to develop two-way contingency tables (preceding *versus* succeeding behavioural states) and calculate the number of transitions between the behavioural states in both control and interaction conditions. Foraging, milling and travelling behaviours are likely to be affected by the previous interaction up to 15min following the departure of the vessel [9]. Based on this assumption, post-interaction sequences of 15min immediately following the departure of interacting vessel(s) were added to the interaction sequences for further analyses.

**Table 2:** Different approaches of Markov chain analysis.

**Type of approach**	**Conservative**				**Less conservative**			
**Scenario**	**1**		**2**>		**3**		**4**	
	S_1_		S_1_		S_1_		S_1_	
								
	S_2_		S_2_		S_2_		S_2_	
								
	S_3_		S_3_		S_3_		S_3_	
		←				←		
	S_4_		S_4_	←	S_4_		S_4_	←
								
**3min samples**	S_5_		S_5_		S_5_		S_5_	
								
	S_6_		S_6_		S_6_		S_6_	
								
	S_7_		S_7_	→	S_7_		S_7_	→
		→				→		
	S_8_		S_8_		S_8_		S_8_	
								
	S_9_		S_9_		S_9_		S_9_	
								
	S_10_		S_10_		S_10_		S_10_	
**Discarded samples**	S_4_, S_8_		S_4_, S_7_		S_8_		S_8_	
**Control chains**	S_1_S_2_S_3_–S_9_S_10_		S_1_S_2_S_3_–S_8_S_9_S_10_		S_1_S_2_–S_9_S_10_		S_1_S_2_–S_9_S_10_	
**Interaction chains**	S_5_S_6_S_7_		S_5_S_6_		S_3_S_4_S_5_S_6_S_7_		S_3_S_4_S_5_S_6_S_7_	

The leftward and rightward arrows indicate the vessel arrival and departure, respectively. **Conservative approach—** *Scenario 1*: Vessel arrives/departs between samples S3 and S4, and S7 and S8, respectively. Samples S4 and S8 following the vessel arrival/departure are discarded. *Scenario 2*: Vessel arrives/departs during samples S4 and S7, respectively. S4 and S7 are discarded. **Less conservative approach—** *Scenario 3*: Vessel arrives and departs between S3 and S4, and S7 and S8, respectively. S3 is considered affected by the vessel arrival. Sample S8 following the vessel departure is discarded. *Scenario 4*: Vessel arrives/departs during S4 and S7, respectively. Sample S3 preceding the vessel arrival is considered affected. Sample S8 following the vessel departure is discarded.

Following the Perron-Frobenius theorem [80], the behavioural budget (i.e. the proportion of time dolphins engaged in each behavioural state [9, 10, 46]) under control and interaction conditions was approximated by the left eigenvector of the dominant eigenvalue of the transition matrices using the Excel add-in PopTools (Version 3.0.3, CSIRO: www.cse.csiro.au/poptools/). Differences between control and interaction behavioural budgets were tested with a binomial Z-test for proportions [81] and 95% confidence intervals (CI) were calculated.

To assess changes in behavioural states due to vessel presence, transition probabilities, from the immediately preceding to the succeeding behavioural state, were calculated for the control and interaction chains separately by [10]:

$$p_{ij}=a_{ij}/\sum_{j=1}^{n}a_{ij},\sum_{j=1}^{n}p_{ij}=1$$

where *i* is the preceding behavioural state, *j* is the succeeding behavioural state, *a_ij_* is the number of transitions observed from behavioural state *i* to *j*, *p_ij_* is the transition probability from *i* to *j* in the Markov chain and *n* is the total number of behavioural states. Control and interaction transition probabilities were compared using a binomial Z-test for proportions [81] and 95% CI were calculated.

To assess the recovery period after disturbance for different behavioural states, the average time (min) it took dolphins to return to each initial behavioural state was calculated and compared between control and interaction conditions, following Stockin *et al*. [9]:

$$E(T_{j})=\frac{1}{\pi_{j}}$$

where (*Tj*) denotes the time (i.e. number of transitions multiplied by the length of each transition unit, i.e. 3min) it takes to return to state *j* given that the dolphins are currently in state *j* and *π* is the steady-state probability of each behaviour in the chain.

Behavioural bout lengths $\bar{t_{ii}}$ were also estimated from the Markov chains, as detailed in Lusseau [10], and compared between control and interaction situations using the Student’s t-test. Pearson’s χ^2^ tests were used to examine any difference in the identified effects while using both sensitivities for the Markov chain analyses. Statistical analyses were performed using the statistical software R 3.0.1 [82].

**Levels of vessel traffic.** During a focal follow, each vessel interacting with dolphins was considered an independent sampling unit. On a broad scale, commercial TV were compared to non-tour vessels (hereafter non-TV, categories b-f, as described previously). Vessel traffic analysis examined the number and type of vessels interacting, separately or simultaneously, with the focal group of dolphins. The duration (min) of the encounters was examined with regards to the maximum time of 90min allowed per vessel, as defined in the commercial tour permits. The number of approaches per vessel was also reported. For each focal group, the overall time dolphins spent in the presence of vessels was estimated and compared according to the type of vessel. When a vessel interacted with a focal group more than once, successive encounters were cumulated, interaction time was summed and compared between vessel types using non-parametric Kruskal-Wallis tests. The speed (kts) of each vessel was recorded every 3min while within 300m of the focal group and compared according to the different types of vessels (Kruskal-Wallis tests). If a vessel encountered a focal dolphin group and attempted to approach and interact more than once with that same group, the second attempt was excluded from the speed analysis to ensure independence across encounters [46].

**Cumulative behavioural budget.** The interaction behavioural budget describes the behaviour of the dolphins during interactions with vessels. Thus, it is an instantaneous measure, which does not take into account the amount of time that dolphins are exposed to interacting vessels throughout the year. To incorporate boat exposure into the behavioural effect on dolphins, the dolphin cumulative behavioural budget (seasonal behavioural budget) was estimated following Lusseau [10] and Christiansen *et al*. [12]:

Cumulative budget=(a×impact budget)+(b×control budget)

where *a* is the proportion of time (relative number of daylight hours per day) that common dolphins spend with interacting vessels (thus following a behavioural budget similar to interaction) and b = 1 - *a* is the proportion of time dolphins spend without interacting vessels (thus following a behavioural budget similar to control). If dolphins had no exposure to interacting vessels, *a* would equal 0, and the cumulative behavioural budget of the dolphins would be the same as the control budget. Conversely, if the dolphins were interacting with vessels throughout all the daylight hours, *a* would equal 1, and the cumulative behavioural budget would be the same as the interaction budget. To test if the dolphin cumulative behavioural budget was significantly different from their control budget, a 2-tailed Z-test for proportions for each behavioural state was used. The effects of vessel traffic intensity on the dolphin cumulative behavioural budget was also investigated by artificially changing *a* from 0 to 100% and testing if the resulting cumulative behavioural budget differed significantly from the control budget [12].

**Effect of swimmers.** The size and composition of the group of dolphins interacting with the vessel while swim activities occurred were monitored from both the TV and the RV, in order to compare the level of compliance of commercial tour operations. The duration (min) of the swim attempts, dolphin behavioural state (Table 1) and dolphin responses to swimmers, as well as the different reasons for ending a swim encounter were examined.

## Results

### Field effort

During the study period, a total of 55 focal follows were undertaken during 7,634min (i.e. 127.2h) and 828.5km of survey effort across 50d aboard the RV. Control and interaction sequences of more than 15min (i.e. composed of a minimum of five transitions) were considered for Markov chain analyses (as per Stockin *et al*. [9]). Regardless of whether in control or interaction conditions, and using the more or less conservative Markov chain approach, only a low number of transitions between resting and socialising and the other behavioural states were observed. Moreover, the low proportion of time spent resting and socialising (less than 13.5%) in the overall behavioural budget of the dolphins precluded the use of those two behavioural states in further analyses. Any transitions containing resting and/or socialising states were therefore omitted and Markov chain analyses were examined taking into account only the three remaining behavioural states, i.e. foraging, milling and travelling (Table 3).

**Table 3:** Number and duration (mean and range in min) of sequences and number of behavioural transitions during control scenarios (presence of the research vessel only) and during interaction scenarios (when in the presence of other vessels).

**Type of approach**	**Conservative**	**Less conservative**
*Control conditions*		
Number of sequences	38.6% (n = 34)	39.8% (n = 33)
Duration of sequences	49.8 (15.0–279.0)	50.8 (15.0–279.0)
Number of transitions	60.2% (n = 564)	60.1% (n = 559)
		
*Interaction conditions*		
Number of sequences	61.4% (n = 54)	60.2% (n = 50)
Duration of sequences	20.7 (15.0–81.0)	22.3 (15.0–87.0)
Number of transitions	39.8% (n = 373)	39.9% (n = 371)

Calculated and presented considering three behavioural states (foraging, milling and travelling) and using the conservative and less conservative approaches, respectively.

### Effect of boat interactions

**Figure 2:**
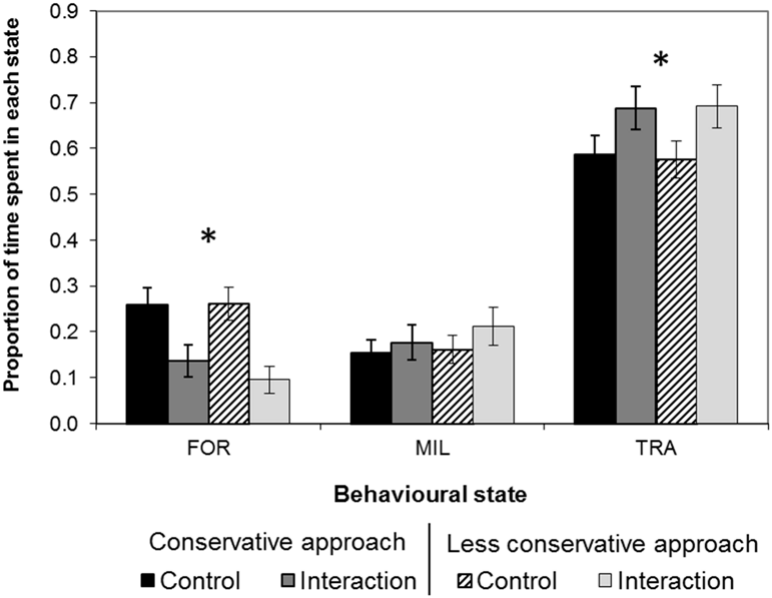
Effect of vessel interactions on the behavioural budget of common dolphins in the Bay of Plenty. Proportion of time spent in each behavioural state in the presence and absence of interacting vessels. Error bars represent 95% confidence intervals. Significant differences (p<0.05) between control (solid or striped black bars) and interaction scenarios (light and dark grey bars) are denoted by an (*). Results are shown following the conservative and less conservative approaches. Note: FOR = foraging, MIL = milling, TRA = travelling.

Following the conservative approach and under control conditions, common dolphins spent the majority (58.9%, n = 352) of their time travelling. Foraging represented an important proportion of their behaviour (26.8%, n = 160), while milling accounted for only half of that time (14.4%, n = 86). There was no significant difference to this pattern while following the less conservative approach (Pearson’s χ^2^: χ^2^ = 0.12, df = 2, p>0.05). The behaviour of common dolphins differed in the presence of vessels (Fig. 2). Travelling increased by 10.1% (95% CI: 3.7–16.4%, z = -3.12, p = 0.002) or by 11.7% (95% CI: 5.3–18.0%, z = -3.60, p<0.001), while foraging decreased by 12.4% (95% CI: 7.1–17.8%, z = 4.56, p<0.001) or by 16.7% (95% CI: 11.5–21.9%, z = 6.30, p<0.001), according to the conservative and less conservative approach, respectively.

**Figure 3:**
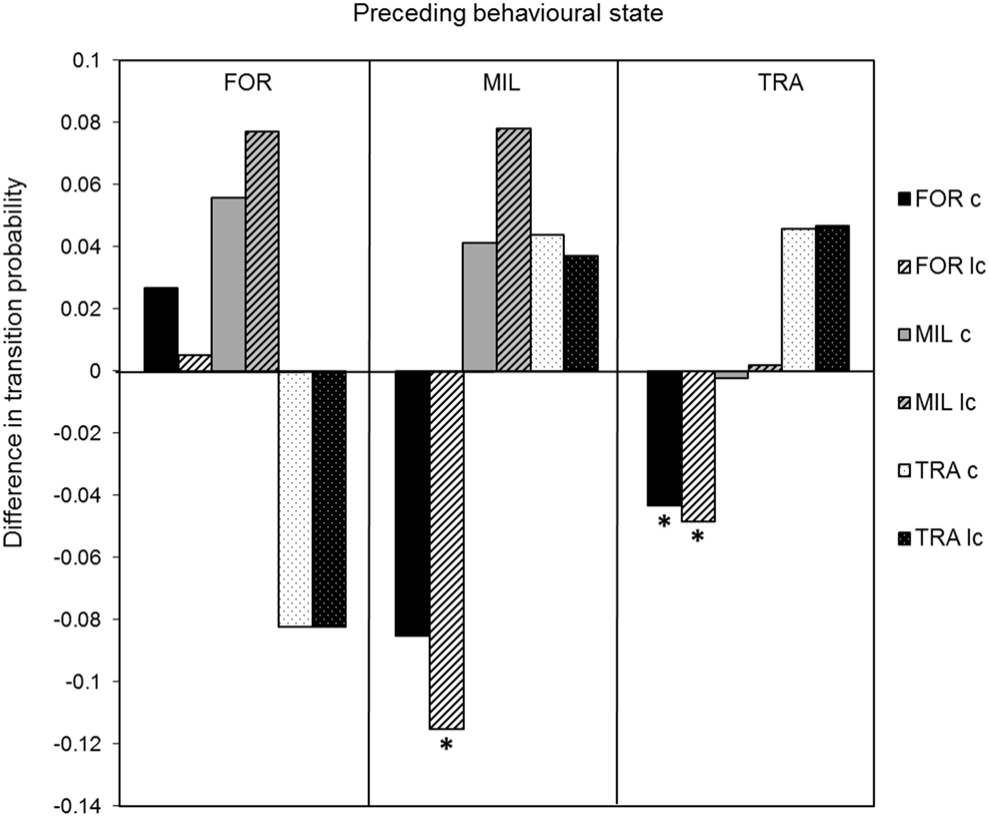
Effect of vessel presence on transitions between behavioural states of common dolphins, based on differences in transition probabilities(*p_ij_*_(interaction)_-*p_ij(_*_control)_). A negative value means that the behavioural transition of the control chain is superior to that of the interaction chain. The graph is composed of three parts, one for each preceding state, separated by vertical lines. In each part, bars correspond to succeeding behavioural states (see legend). Transitions with a significant difference (p<0.05) are marked by an (*). Results shown after following the conservative approach (c) and the less conservative approach (lc). Note: FOR = foraging, MIL = milling, TRA = travelling.

The temporal dependence between behavioural states was also affected by vessel presence. The transition from travelling to foraging significantly decreased by 67.9% (Z-test: z = 2.47, p<0.05) when using the conservative approach (Fig. 3). Based on the less conservative approach, the same transition decreased more (74.7%, Z-test: z = 2.78, p<0.05) and transition from milling to foraging significantly decreased by 67.5% (Z-test: z = 2.41, p<0.05). Moreover, once disrupted, foraging dolphins took longer to return to this state, with an increase of 91.4% or 175.2%, from 11.5min to 22.1min or to 31.5min, for the conservative or less conservative approach, respectively (Table 4). Time taken to return to milling and travelling decreased by 13.3% from 19.6min to 17.0min and by 14.6% from 5.1min to 4.4min, respectively, in the presence of interacting vessels when using the conservative approach (Table 4). Using the less conservative approach, time to return to milling and travelling was shortened (time decreased by 23.6% from 18.5min to 14.1min and by 16.8% from 5.2min to 4.3min, respectively, Table 4). The average length of behavioural bouts significantly varied when vessels were present (Table 5). Bout length increased by 12.2% for foraging dolphins (95% CI: 0.36–0.64; t = -7.20, df = 202, p<0.001) when using the conservative approach, while the less conservative approach found no difference. For travelling dolphins, bout length increased by 55.9% (95% CI: 4.34–4.44; t = -168.33, df = 573, p<0.001) or by 54.2% (95% CI: 4.04–4.14; t = -157.31, df = 561, p<0.001), for the conservative and less conservative approach, respectively. Similarly, during interactions, the duration of milling bouts increased by 11.9% (95% CI: 0.16–0.46; t = -4.05, df = 156, p<0.001) or by 26.0% (95% CI: 0.54–0.82; t = -9.43, df = 166, p<0.001), for the conservative and less conservative approach, respectively.

**Table 4:** Probability of being in a particular behavioural state (π_j_), number of 3min time units (E(*T*_*j*_)) and amount of time (min) required to return to initial behavioural states during control scenarios (presence of the research vessel only) and during interaction scenarios (when in the presence of other vessels).

		**Control**			**Interaction**	
**Behaviour**	**π_j_**	**E(*T_j_*)**	**Time (min)**	**π_j_**	**E(*T_j_*)**	**Time (min)**
**Foraging**	0.26 / 0.26	3.8 / 3.8	11.5 / 11.5	0.14 / 0.10	7.4 / 10.5	22.1 / 31.5
**Milling**	0.15 / 0.16	6.5 / 6.2	19.6 / 18.5	0.18 / 0.21	5.7 / 4.7	17.0 / 14.1
**Travelling**	0.59 / 0.58	1.7 / 1.7	5.1 / 5.2	0.69 / 0.69	1.5 / 1.4	4.4 / 4.3

Calculated and presented using the conservative/less conservative approaches, respectively.

**Table 5:** Average bout length ($\bar{t_{ii}}$ ) during control (presence of the research vessel only) and interaction scenarios (presence of other vessels).

**Behaviour**	**Control $\bar{t_{ii}}$**	**Interaction $\bar{t_{ii}}$**
**Foraging**	4.05 / 4.03	4.55 / 4.11
**Milling**	2.58 / 2.65	2.89 / 3.33
**Travelling**	7.86 / 7.53	12.25 / 11.62

Numbers represent the conservative/less conservative estimates, respectively.

### Levels of vessel traffic

Interactions between vessels and dolphins were monitored during 256 surveys undertaken from aboard the TV and during the 35 focal follows monitored aboard the RV (i.e. 186 vessel-common dolphin interactions). Out of the 7,634min (i.e. 127.2h) of focal follows recorded by the RV, common dolphins were observed in the presence of vessels during 21.0% of the time (1,604min, i.e. 26.7h), of which 6.0% (459min, i.e. 7.7h) was with TV only, 1.7% (133min, i.e. 2.2h) with non-TV only and 13.3% (1,012min, i.e. 16.9h) with both types of vessels. Overall, common dolphin groups spent significantly more time in the presence of TV (median = 45min, IQR = 38.5min, n = 11) than in the presence of non-TV (median = 9min, IQR = 7.3min, n = 8, Kruskal-Wallis: h = 5.17, df = 1, p<0.05). Similarly, when assessing interactions per vessel, TV spent significantly more time with common dolphins (median = 37min, IQR = 33.5min, n = 23) than non-TV (median = 1min, IQR = 4min, n = 139, Kruskal-Wallis: h = 55.31, df = 1, p<0.001). Interactions monitored from aboard the TV lasted between one and 148min (median = 40.5min, IQR = 38.8min, n = 256), exceeding the 90min time restriction specified in the permit regulations during 14.8% of encounters (n = 38).

Generally, between one and three vessels interacted with a focal group of common dolphins (80.0% of the focal follows, n = 28), although a maximum of 61 vessels, including TV, were observed approaching dolphins inside Tauranga harbour during the course of this study, in contravention of the permit regulations. Moreover, simultaneous interactions (n = 29), where one vessel interacting with dolphins was joined by others, were relatively frequent (42.9% of focal follows, n = 15) and the majority of interactions (75.9%, n = 22) involved two or three vessels. While this was in compliance with the New Zealand MMPR [65], it was not unusual (24.1%, n = 7) to observe four or more vessels interacting with the same group of dolphins, in breach of the regulations [65]. This included one occasion when a TV arrived after two TV and two non-TV were already within 300m of the dolphins. TV primarily approached dolphins once (88.6%, n = 39), but were occasionally observed interacting twice with the same focal group (11.4%, n = 5). Similarly, non-TV mainly approached dolphins once (90.1%, n = 128), although some did approach the same focal group twice (9.2%, n = 13) or up to four times (7.0%, n = 1).

Vessel types travelled at significantly different speeds (Kruskal-Wallis: h = 76.08, df = 5, p<0.001) when within 300m of dolphin groups. Non-motorised craft interacted with the dolphins below the “non-wake” speed (median = 2.5kts, IQR = 3.5kts, n = 12). Motorised commercial vessels (median = 7.0kts, IQR = 7.0kts, n = 19) and motorised recreational launches (median = 10.0kts, IQR = 5.8kts, n = 20) typically passed within 300m of dolphins without altering either their course or speed. Motorised recreational trailer-launched vessels (median = 10.0kts, IQR = 14.0kts, n = 71) and motorised personal craft (median = 15.0kts, IQR = 11.0kts, n = 3) showed a wide range of speeds, reacting to dolphin presence via sudden altering of course and/or speed. Commercial TV travelled around 5kts (median = 5.5kts, IQR = 3kts, n = 275) but were observed 51.3% of the time (n = 141) travelling over 5kts within 300m of dolphins, in breach of the regulations [65].

### Cumulative behavioural budget

**Figure 4:**
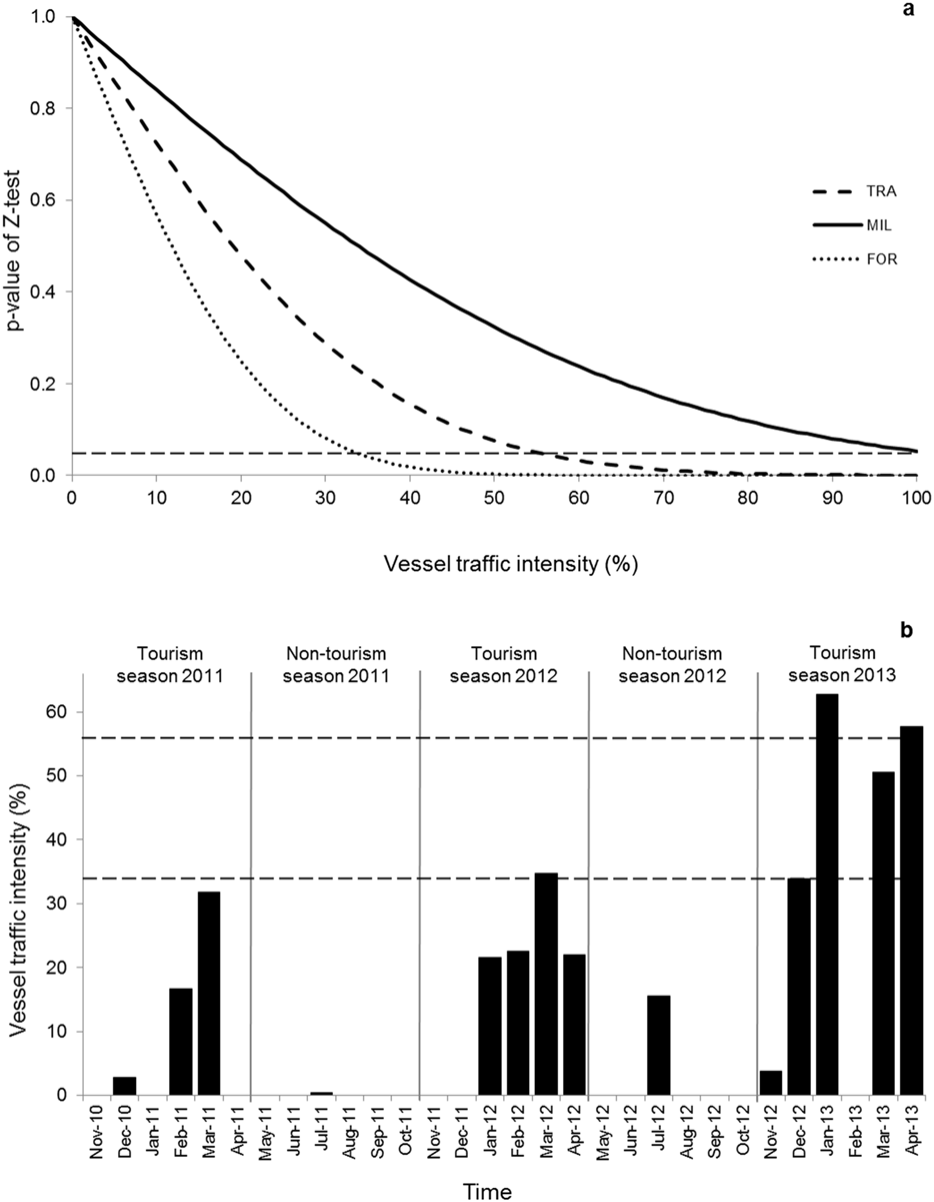
Effect of vessel traffic intensity on dolphin behaviour. a) P-values of the difference between the cumulative behavioural budget and the control behavioural budget for common dolphin activity. The proportion of time dolphins spent with interacting vessels was artificially varied from 0 to 100%. Each curve corresponds to different behavioural states (FOR = foraging, MIL = milling, TRA = travelling). The horizontal dashed line represents the statistical level of significance (p<0.05) b) Vessel traffic intensity throughout the study period (November 2010 to April 2013). The horizontal dashed lines represent 34.0 and 56.0% of traffic intensity above which the cumulative foraging and travelling behaviours, respectively, are significantly affected. The vertical lines separate the tourism and non-tourism seasons.

We found that a vessel traffic intensity exceeding 34.0 and 56.0% would significantly affect common dolphin cumulative foraging and travelling behaviours, respectively (Fig. 4a). Therefore, the overall vessel traffic intensity of 21.0% does not significantly affect dolphin cumulative behavioural budget over time (Fig. 4a). However, when looking at a finer temporal scale, these critical levels were reached temporarily during the peak tourism season of 2012 and 2013 (Fig. 4b).

### Swimming with the dolphins

Overall, 26 swim attempts were monitored during 12 swim encounters from the RV. Additionally, 67 swim attempts during 25 swim encounters were monitored from aboard the TV. During the 12 swim encounters with common dolphins monitored from the RV, swimmers were primarily deployed by the TV (83.3%, n = 10), although recreational boats (i.e. motorised trailer-launched vessels) dropped single swimmers on two independent occasions (16.7%).

Swims lasted only 5.2min on average (SD = 3.9min, n = 61), with the majority (59.0%, n = 36) lasting less than 5min and only a small proportion (11.5%, n = 7) lasting more than 10min. When monitored from aboard the TV, the majority (77.1%, n = 27) of swims occurred with small dolphin groups (1–10 individuals) containing only adults. Twenty percent (n = 7) of the swims occurred with larger groups (11–30 individuals) containing adults and juveniles and on one occasion calves, in contravention to the New Zealand MMPR [65]. Moreover, one swim encounter (2.9%) occurred with a group larger than 200 individuals which contained all age classes, in breach of the regulations [65]. Conversely, out of the 12 swim encounters monitored from the RV, calves were observed in the group during 50.0% of the swims (TV n = 5, recreational boat n = 1), in breach of the New Zealand MMPR [65]. Juveniles were present during all 12 swim encounters.

Swimmers were placed in the water when common dolphins were travelling (34.0%, n = 17), foraging (26.0%, n = 13), socialising (22.0%, n = 11) or milling (18.0%, n = 9). When swimmers were present in the water, the proportion of encounters where dolphins did not change their behavioural state (i.e. neutral) was significantly higher (56.8%, n = 21, χ^2^ = 11.73, df = 2, p<0.05), compared to only 32.4% (n = 12) and 10.8% (n = 4) of observations where dolphins approached or avoided the swimmers, respectively. Swim encounters with common dolphins ended 70.1% of the time (n = 47) because of skipper decision, 28.4% (n = 19) of the time because of loss of sight of dolphins and 1.5% of the time (n = 1) because of calf presence. Furthermore, only 53.7% (n = 39) of the monitored swimmers actually reported observing common dolphins subsurface.

## Discussion

In the history of marine mammal exploitation, tourism has often been considered positively compared to lethal whaling activities [83, 84]. In addition, watching free-ranging dolphins is becoming a popular alternative to watching dolphins in captivity [85, 86, 87]. However, effects of commercial tourism activities on marine mammals are becoming difficult to ignore. Since the 1990s, research has raised concerns about the effects of commercial tourism on marine mammal behaviour, reporting various changes in the behaviour of numerous coastal species (e.g. [8, 9, 11, 12, 13, 14, 45]). Regardless of either Markov approach applied, our study provides further evidence that commercial tourism induces significant changes in the behaviour of common dolphins using open oceanic waters. More specifically, the presence of interacting vessels affected the behavioural budget of common dolphins, which spent significantly less time foraging. Once disrupted, dolphins took at least twice as long to return to foraging when compared to control conditions. Furthermore, the probability of starting to forage while engaged in travelling decreased by two thirds. Conversely, dolphins increased their foraging bout length in the presence of interacting vessels (following the conservative approach). Given foraging tactics used by common dolphins include cooperative herding of the prey [88, 89, 90, 91], it is possible that the behavioural changes of some individuals, as a result of approaching vessels, could compromise the success of the overall foraging event. Manoeuvring a vessel through a group of dolphins, as it has been observed, may separate individuals within the dolphin group, disperse the prey and/or affect dolphin communication because of vessel underwater noise [44, 92, 93]. In all scenarios, dolphins would presumably have to re-establish group cohesion and/or communication in order to successfully capture their prey, ultimately resulting in both increased time between foraging bouts and energy expenditure. Thus, our findings indicate that common dolphin foraging behaviour is significantly affected by the presence of interacting vessels in the central and eastern BOP.

Foraging is a critical component for any predator and disruption to this behaviour can potentially result in energy intake reductions that can have long-term implications if the population is limited by resource availability [35, 78]. In an environment like the BOP, where prey resources are widespread and unpredictable in distribution [63], as demonstrated by the large proportion of time dolphins spend travelling in search of prey patches [63], interactions with vessels are likely to lead to a reduction in the overall energy acquisition. Notably, the majority of dolphin tourism in the region occurs during the austral summer, during the peak calving season [76, 94], when there is a higher occurrence of common dolphins closer to the shore [62]. Our results show that the cumulative time spent foraging and travelling were significantly affected in the tourism seasons of 2012 and 2013. Although the consequences of reduced feeding for nursing groups remain unclear, it is likely to have bigger effects on pregnant and lactating females [95]. Indeed, in order to cope with increased energetic expenses, pregnant and lactating females have been shown to change their diet for more energy-rich prey so as to maximise their rate of energy intake or meet their nutritional requirements [95, 96, 97]. It has also been suggested that different boat avoidance strategies exist between male and female dolphins, likely as a consequence of different energetic demands [98]. Therefore, disrupting the foraging behaviour of females and immature dolphins is likely to add extra physiological constraints to these individuals and could potentially reduce their reproductive success and negatively affect population dynamics on a long-term basis [98].

Similarly to bottlenose dolphins [99, 100], movements of some common dolphins across neighbouring regions in the North Island have been confirmed [101]. Indeed, several individuals identified in the Hauraki Gulf have previously or subsequently been observed in the Bay of Islands or in the BOP, 200km further north and south east, respectively [102]. This further highlights the potential risk of cumulative effects across all regions within the home range of the population. We therefore highlight the importance of developing management strategies that engage neighbouring regions. Until the common dolphin population has been reassessed and potential cumulative effects have been quantified across the whole home range, we recommend a moratorium on further permits targeting the species in the central-eastern and north-eastern North Island waters.

In the North Island, commercial swimming with common dolphins is permitted in the Bay of Islands, Hauraki Gulf and in the BOP. However, common dolphins seem to be less receptive to this activity compared to other species. For example, common dolphins in the BOP infrequently approached swimmers (32.4% of encounters), as previously observed in Mercury Bay (20.5% [8]) and in the Bay of Islands (24.1% [61]). Thus, swim encounters where dolphins actively approach swimmers are less frequent for common dolphins than conspecifics (bottlenose, Hector’s and dusky dolphins, *Lagenorhynchus obscurus*) targeted by swim-with-dolphins operators in New Zealand [23, 103, 104, 105], all of which approached swimmers during more than 50.0% of swim attempts. Similarly to Bay of Islands and Mercury Bay [8, 61], swimmers typically spent only 5min in the water, compared to longer durations with dusky or Hector’s dolphins, which lasted 9.1 and 10–18.8min, respectively [23, 106]. Moreover, only half of the swimmers questioned in our study reported having actually observed common dolphins subsurface. While this relatively low level of success could be explained by water turbidity or lack of swimmer confidence, dolphin group size and behavioural state are likely a key influence on swim success, with dolphins being more interactive when in larger socialising groups compared to when travelling or milling [8]. Finally, the manoeuvring of the TV and change of speed during swim encounters (slowing down/stopping to place people in the water and then pursuing the dolphins) might also explain the low dolphin interest.

This study also highlighted non-compliance to some permit conditions and/or regulations (e.g. area of operation, speed and number of vessels interacting with a single group, maximum time permitted interacting with the dolphins and swimming with calves). Compliance also varied when recorded from aboard the RV and TV and could be explained by tour operators reacting to the presence of a researcher aboard their vessels and adhering more closely to the regulations. Alternatively, the researcher was likely to act as an independent observer alerting the skipper about breaches of regulation (e.g. warning about the presence of calves while not focusing on the swimmers). Adherence to management regulations has not only been shown to reduce effects of vessel interactions on dolphin behaviour [107], but also increase the probability of having an interaction with a dolphin group. For example, dolphins have been shown to avoid high speed vessels and conversely associate for longer periods of time with slower craft (e.g. kayaks or sailing vessels [46]). Besides changes in dolphin behaviour [108, 109], high speed driving can also result in an elevated risk of collision which can be fatal [17, 60, 110, 111].

In New Zealand, common dolphins are currently classified as ‘not threatened’ [52] and are still lacking a species-specific Marine Mammal Action Plan [54], despite numerous threats [53] which include pollution [58], fisheries by-catch [59] and vessel collisions [60]. In the light of our results and previous studies, tourism has now clearly been identified as an additional human induced threat, as viewing and swimming activities significantly affect the species behaviour in various regions around New Zealand [8, 9]. Moreover, its cumulative effects across dolphin home range are likely to exacerbate identified impacts. As previously described (e.g. [46, 107]), dolphins are likely to use the area until the costs of tolerance exceed the benefits of remaining in that habitat. In species such as dolphins, the long-term effects of tourism activities can take decades to detect [112, 113]. Common dolphins are therefore unlikely to immediately discontinue use the BOP waters, despite facing human disturbance (e.g. recreational vessel traffic, commercial pressure, etc), thus regular monitoring of the local population is required.

## Conclusions

This study shows that tourism activities on common dolphins in open oceanic waters can be as detrimental as in inshore shallow coastal seas. Overall, interacting vessels significantly affected a biologically important behaviour, namely foraging. The magnitude of this effect is a cause for concern given its impact on common dolphin cumulative behavioural budget during the peak tourism season, which is also the calving and breeding season for this species in New Zealand waters. Not only is it the busiest period for commercial tourism activities, but recreational vessel traffic is at its highest and adds considerable time to the interaction with the dolphins in addition to the commercial vessels. Therefore, future growth in commercial tourism activities as well as recreational interactions in this area need careful consideration. Given that non-compliance to the regulations (permit conditions and New Zealand MMPR [65]) was recorded, appropriate conservation management is recommended and should further encompass neighbouring regions so as to consider cumulative effects of vessel interactions across the home range of the population.

## References

[1] Williams R, Bain DE, Smith JC, Lusseau D (2009) Effects of vessels on behaviour patterns of individual southern resident killer whales *Orcinus orca* . Endangered Species Research 6: 199–209. 10.3354/esr00150

[2] Lusseau D (2004) The hidden cost of tourism: Detecting long-term effects of tourism using behavioral information. Ecology and Society 9: 2.

[3] Bejder L, Samuels A, Whitehead H, Gales N, Mann J, et al (2006) Decline in relative abundance of bottlenose dolphins exposed to long-term disturbance. Conservation Biology 20: 1791–1798. 10.1111/j.1523-1739.2006.00540.x 17181814

[4] Orams M (2004) Why dolphins may get ulcers: Considering the impacts of cetacean-based tourism in New Zealand. Tourism in Marine Environments 1: 17–28.

[5] Scarpaci C, Parsons ECM (2013) Recent advances in whale-watching research: 2012–2013. Journal of Cetacean Research and Management SC/65a/WW01: 1–18.

[6] Parsons ECM (2012) The negative impacts of whale-watching. Journal of Marine Biology 2012: 1–9. 10.1155/2012/807294

[7] Higham JES, Bejder L, Williams R (2014) Whale-watching, sustainable tourism and ecological management. Higham JES, Bejder L, Williams R, editors. Cambridge, UK: University Press 418 p.

[8] Neumann DR, Orams MB (2006) Impacts of ecotourism on short-beaked common dolphins (*Delphinus delphis*) in Mercury Bay, New Zealand. Aquatic Mammals 32: 1–9. 10.1578/AM.32.1.2006.1

[9] Stockin KA, Lusseau D, Binedell V, Wiseman N, Orams MB (2008) Tourism affects the behavioural budget of the common dolphin *Delphinus* sp. in the Hauraki Gulf, New Zealand. Marine Ecology Progress Series 355: 287–295.

[10] Lusseau D (2003) Effects of tour boats on the behavior of bottlenose dolphins: Using Markov chains to model anthropogenic impacts. Conservation Biology 17: 1785–1793. 10.1111/j.1523-1739.2003.00054.x

[11] Stensland E, Berggren P (2007) Behavioural changes in female Indo-Pacific bottlenose dolphins in response to boat-based tourism. Marine Ecology Progress Series 332: 225–234. 10.3354/meps332225

[12] Christiansen F, Lusseau D, Stensland E, Berggren P (2010) Effects of tourist boats on the behaviour of Indo-Pacific bottlenose dolphins off the south coast of Zanzibar. Endangered Species Research 11: 91–99. 10.3354/esr00265

[13] Stamation KA, Croft DB, Shaughnessy PD, Waples KA, Briggs SV (2010) Behavioral responses of humpback whales (*Megaptera novaeangliae*) to whale-watching vessels on the southeastern coast of Australia. Marine Mammal Science 26: 98–122. 10.1111/j.1748-7692.2009.00320.x

[14] Steckenreuter A, Moller L, Harcourt R (2012) How does Australia’s largest dolphin-watching industry affect the behaviour of a small and resident population of Indo-Pacific bottlenose dolphins? Journal of Environmental Management 97: 14–21. 10.1016/j.jenvman.2011.11.002 22325578

[15] Laist DW, Knowlton AR, Mead JG, Collet AS, Podesta M (2001) Collisions between ships and whales. Marine Mammal Science 17: 35–75. 10.1111/j.1748-7692.2001.tb00980.x

[16] International Whaling Commision (2014) Report of the Sub-Committee on whalewatching. Journal of Cetacean Research and Management 15 (Suppl): 380–392.

[17] Dwyer SL, Kozmian-Ledward L, Stockin KA (2014) Short-term survival of severe propeller strike injuries and observations on wound progression in a bottlenose dolphin. New Zealand Journal of Marine and Freshwater Research 48: 1–9. 10.1080/00288330.2013.866578

[18] Martinez E, Orams MB, Pawley MDM, Stockin KA (2012) The use of auditory stimulants during swim encounters with Hector’s dolphins (*Cephalorhynchus hectori hectori*) in Akaroa Harbour, New Zealand. Marine Mammal Science 28: E295–E315. 10.1111/j.1748-7692.2011.00528.x

[19] Martinez E, Orams M (2011) Kia angi puku to hoe I te wai: Ocean noise and tourism. Tourism in Marine Environments 7: 191–202. 10.3727/154427311X13195453162895

[20] Lachmuth CL, Barrett-Lennard LG, Steyn DQ, Milsom WK (2011) Estimation of southern resident killer whale exposure to exhaust emissions from whale-watching vessels and potential adverse health effects and toxicity thresholds. Marine Pollution Bulletin 62: 792–805. 10.1016/j.marpolbul.2011.01.002 21276987

[21] Constantine R (2001) Increased avoidance of swimmers by wild bottlenose dolphins (*Tursiops truncatus*) due to long-term exposure to swim-with-dolphin tourism. Marine Mammal Science 17: 689–702. 10.1111/j.1748-7692.2001.tb01293.x

[22] Filby NE, Stockin KA, Scarpaci C (2014) Long-term responses of Burrunan dolphins (*Tursiops australis*) to swim-with dolphin tourism in Port Phillip Bay, Victoria, Australia: A population at risk. Global Ecology and Conservation 2: 62–71. 10.1016/j.gecco.2014.08.006

[23] Martinez E, Orams M, Stockin KA (2011) Swimming with an endemic and endangered species: Effects of tourism on Hector’s dolphins in Akaroa harbour, New Zealand. Tourism Review International 14: 99–115. 10.3727/154427211X13044361606379

[24] Samuels A, Bejder L (2004) Chronic interaction between humans and free-ranging bottlenose dolphins near Panama City Beach, Florida, USA. Journal of Cetacean Research and Management 6: 69–77.

[25] Rafic M (1999) Report of the dolphin feeding program at Monkey Mia Shark Bay, Western Australia. Report of the Scientific Comittee, Annex J. Appendix 2. Journal of Cetacean Research and Management 1 (Suppl): 230–232.

[26] Orams MB (2002) Feeding wildlife as a tourism attraction: A review of issues and impacts. Tourism Management 23: 281–293. 10.1016/S0261-5177(01)00080-2

[27] Donaldson R, Finn H, Calver M (2010) Illegal feeding increases risk of boat-strike and entanglement in bottlenose dolphins in Perth, Western Australia. Pacific Conservation Biology 16: 157–161.

[28] Mann J, Connor RC, Barre LM, Heithaus MR (2000) Female reproductive success in bottlenose dolphins (*Tursiops* sp.): Life history, habitat, provisioning, and group-size effects. Behavioral Ecology 11: 210–219. 10.1093/beheco/11.2.210

[29] Foroughirad V, Mann J (2013) Long-term impacts of fish provisioning on the behavior and survival of wild bottlenose dolphins. Biological Conservation 160: 242–249. 10.1016/j.biocon.2013.01.001

[30] Orams MB, Hill GJE, Baglioni AJ (1996) “Pushy” behavior in a wild dolphin feeding program at Tangalooma, Australia. Marine Mammal Science 12: 107–117. 10.1111/j.1748-7692.1996.tb00308.x

[31] Smith H, Samuels A, Bradley S (2008) Reducing risky interactions between tourists and free-ranging dolphins (*Tursiops* sp.) in an artificial feeding program at Monkey Mia, Western Australia. Tourism Management 29: 994–1001. 10.1016/j.tourman.2008.01.001

[32] Waltzek TB, Cortes-Hinojosa G, Wellehan JFX, Gray GC (2012) Marine mammal zoonoses: A review of disease manifestations. Zoonoses and Public Health 59: 521–535. 10.1111/j.1863-2378.2012.01492.x 22697432 PMC7477081

[33] International Whaling Commision (2001) Report of the Sub-Committee on whalewatching. Journal of Cetacean Research and Management 3 (Suppl): 297–307.

[34] Boggs CL (1992) Resource allocation: Exploring connections between foraging and life-history. Functional Ecology 6: 508–518. 10.2307/2390047

[35] Williams R, Lusseau D, Hammond PS (2006) Estimating relative energetic costs of human disturbance to killer whales (*Orcinus orca*). Biological Conservation 133: 301–311. 10.1016/j.biocon.2006.06.010

[36] Lusseau D, Bain DE, Williams R, Smith JC (2009) Vessel traffic disrupts the foraging behavior of southern resident killer whales *Orcinus orca* . Endangered Species Research 6: 211–221. 10.3354/esr00154

[37] Christiansen F, Rasmussen MH, Lusseau D (2014) Inferring energy expenditure from respiration rates in minke whales to measure the effects of whale watching boat interactions. Journal of Experimental Marine Biology and Ecology 459: 96–104. 10.1016/j.jembe.2014.05.014

[38] Christiansen F, Rasmussen M, Lusseau D (2013) Whale watching disrupts feeding activities of minke whales on a feeding ground. Marine Ecology Progress Series 478: 239–251. 10.3354/meps10163

[39] O’Connor S, Campbell R, Cortez H, Knowles T (2009) Whale watching worldwide: Tourism numbers, expenditures and expanding economic benefits. Yarmouth Port, MA, USA: International Fund for Animal Welfare 295 p.

[40] Hoyt E (2001) Whale watching 2001—Worldwide tourism numbers, expenditures and expanding socioeconomic benefits. Yarmouth Port, MA, USA: International Fund for Animal Welfare & United Nations Environmental Program 158 p.

[41] International Fund for Animal Welfare (2005) The growth of the New Zealand whale watching industry. Surry Hills: Prepared by Economists@Large and Associates 26 p.

[42] Hollingworth K (2001) Demography, dispersion, and the effect of human disturbance of New Zealand sea lions (*Phocarctos hookeri*) at Surat Bay, South Otago, New Zealand Postgraduate Diploma in Science, University of Otago 52 p.

[43] Constantine R, Brunton DH, Dennis T (2004) Dolphin-watching tour boats change bottlenose dolphin (*Tursiops truncatus*) behaviour. Biological Conservation 117: 299–307. 10.1016/j.biocon.2003.12.009

[44] Guerra M, Dawson SM, Brough TE, Rayment WJ (2014) Effects of boats on the surface and acoustic behaviour of an endangered population of bottlenose dolphins. Endangered Species Research 24: 221–236. 10.3354/esr00598

[45] Arcangeli A, Crosti R (2009) The short-term impact of dolphin-watching on the behavior of bottlenose dolphins (*Tursiops truncatus*) in Western Australia. Journal of Marine Animals and their Ecology 2: 3–9.

[46] Martinez E (2010) Responses of South Island Hector’s dolphins (*Cephalorhynchus hectori hectori*) to vessel activity (including tourism operations) in Akaroa Harbour, Banks Peninsula, New Zealand PhD Thesis, Massey University 415 p.

[47] Courbis S, Timmel G (2009) Effects of vessels and swimmers on behavior of Hawaiian spinner dolphins (*Stenella longirostris*) in Kealake’akua, Honaunau, and Kauhako bays, Hawai’i. Marine Mammal Science 25: 430–440. 10.1111/j.1748-7692.2008.00254.x

[48] Lundquist D, Gemmell NJ, Würsig B (2012) Behavioural responses of dusky dolphin groups (*Lagenorhynchus obscurus*) to tour vessels off Kaikoura, New Zealand. Plos One 7: e41969 10.1371/journal.pone.0041969 22844536 PMC3402466

[49] Hammond PS, Bearzi G, Bjørge A, Forney K, Karczmarski L, et al (2008) Delphinus delphis. The IUCN Red List of Threatened Species. Version 2014.2 Available: www.iucnredlist.org. Accessed 04 November 2014.

[50] Hammond PS, Bearzi G, Bjørge A, Forney K, Karczmarski L, et al (2008) Delphinus capensis. The IUCN Red List of Threatened Species. Version 2014.2 Available: www.iucnredlist.org. Accessed 04 November 2014.

[51] Townsend AJ, de Lange PJ, Duffy CAJ, Miskelly CM, Molloy J, et al (2008) New Zealand threat classification system manual. Wellington, New Zealand: Department of Conservation 35 p.

[52] Baker CS, Chilvers BL, Constantine R, DuFresne S, Mattlin RH, et al (2010) Conservation status of New Zealand marine mammals (suborders Cetacea and Pinnipedia), 2009. New Zealand Journal of Marine and Freshwater Research 44: 101–115. 10.1080/00288330.2010.482970

[53] Stockin KA, Orams M (2009) The status of common dolphins (*Delphinus delphis*) within New Zealand waters. Journal of Cetacean Research and Management SC/61/SM20: 1–13.

[54] Suisted R, Neale D (2004) Department of Conservation Marine Mammal Action Plan for 2005–2010. Wellington, New Zealand: Department of Conservation 89 p.

[55] Bearzi G, Politi E, Agazzi S, Bruno S, Costa M, et al (2005) Occurrence and present status of coastal dolphins (*Delphinus delphis* and *Tursiops truncatus*) in the eastern Ionian Sea. Aquatic Conservation-Marine and Freshwater Ecosystems 15: 243–257. 10.1002/aqc.667

[56] Bearzi G, Bonizzoni S, Agazzi S, Gonzalvo J, Currey RJC (2011) Striped dolphins and short-beaked common dolphins in the Gulf of Corinth, Greece: Abundance estimates from dorsal fin photographs. Marine Mammal Science 27: E165–E184. 10.1111/j.1748-7692.2010.00448.x

[57] Gaskin DE (1992) Status of the common dolphin, *Delphinus delphis*, in Canada. Canadian Field-Naturalist 106: 55–63.

[58] Stockin KA, Law RJ, Duignan PJ, Jones GW, Porter L, et al (2007) Trace elements, PCBs and organochlorine pesticides in New Zealand common dolphins (*Delphinus* sp.). Science of the Total Environment 387: 333–345. 10.1016/j.scitotenv.2007.05.016 17644163

[59] Stockin KA, Duignan PJ, Roe WD, Meynier L, Alley M, et al (2009) Causes of mortality in stranded common dolphin (*Delphinus* sp.) from New Zealand waters between 1998 and 2008. Pacific Conservation Biology 15: 217–227.

[60] Martinez E, Stockin KA (2013) Blunt trauma observed in a common dolphin (*Delphinus* sp.) likely caused by a vessel collision in the Hauraki Gulf, New Zealand. Pacific Conservation Biology 19: 19–27.

[61] Constantine R, Baker CS (1997) Monitoring the commercial swim-with-dolphin operations in the Bay of Islands. Wellington, New Zealand: Department of Conservation 59 p.

[62] Neumann DR (2001) Seasonal movements of short-beaked common dolphins (*Delphinus delphis*) in the north-western Bay of Plenty, New Zealand: Influence of sea surface temperature and El Niño/La Niña. New Zealand Journal of Marine and Freshwater Research 35: 371–374. 10.1080/00288330.2001.9517007

[63] Neumann DR (2001) The activity budget of free-ranging common dolphins (*Delphinus delphis*) in the northwestern Bay of Plenty, New Zealand. Aquatic Mammals 27: 121–136.

[64] Stockin KA, Murphy SN, Duignan PJ, Perrott MJ, Jones GW, et al (2011) Reproductive biology of New Zealand common dolphins (*Delphinus delphis*). In: The 19th Biennial Conference on the Biology of Marine Mammals, Tampa, Florida, USA.

[65] Marine Mammals Protection Regulations (1992) Parliamentary Counsel Office. Available: http://www.legislation.govt.nz/regulation/public/1992/0322/latest/DLM168286.html. Accessed 2014 Nov 4.

[66] Gaborit-Haverkort T, Stockin KA (2011) East coast Bay of Plenty Conservancy: Marine mammal review. East coast Bay of Plenty Conservancy, Department of Conservation, New Zealand 99 p.

[67] Sharples J (1997) Cross-shelf intrusion of subtropical water into the coastal zone of northeast New Zealand. Continental Shelf Research 17: 835–857. 10.1016/S0278-4343(96)00060-X

[68] Stanton BR, Sutton PJH, Chiswell SM (1997) The East Auckland Current, 1994–95. New Zealand Journal of Marine and Freshwater Research 31: 537–549. 10.1080/00288330.1997.9516787

[69] Tilburg CE, Hurlburt HE, O’Brien JJ, Shriver JF (2001) The dynamics of the East Australian Current system: The Tasman Front, the East Auckland Current, and the East Cape Current. Journal of Physical Oceanography 31: 2917–2943. 10.1175/1520-0485(2001)031<2917:TDOTEA>2.0.CO;2

[70] Meissner AM, Orams M, Martinez E, Stockin KA (2014) Effects of commercial tourism activities on bottlenose and common dolphin populations in east coast Bay of Plenty waters. East coast Bay of Plenty Conservancy, New Zealand 117 p.

[71] Altmann J (1974) Observational study of behavior—Sampling methods. Behaviour 49: 227–267. 10.1163/156853974X00534 4597405

[72] Mann J (1999) Behavioral sampling methods for cetaceans: A review and critique. Marine Mammal Science 15: 102–122. 10.1111/j.1748-7692.1999.tb00784.x

[73] Stockin KA, Binedell V, Wiseman N, Brunton DH, Orams MB (2009) Behavior of free-ranging common dolphins (*Delphinus* sp.) in the Hauraki Gulf, New Zealand. Marine Mammal Science 25: 283–301. 10.1111/j.1748-7692.2008.00262.x

[74] Shane SH (1990) Behavior and ecology of the bottlenose dolphin at Sanibel Island, Florida. In: Leatherwood S, Reeves RR, editors. The bottlenose dolphin. San Diego, CA, USA: Academic Press pp. 245–265.

[75] Bearzi G, Notarbartolo-Di-Sciara G, Politi E (1997) Social ecology of bottlenose dolphins in the Kvarnerić (northern Adriatic Sea). Marine Mammal Science 13: 650–668. 10.1111/j.1748-7692.1997.tb00089.x

[76] Stockin KA, Pierce GJ, Binedell V, Wiseman N, Orams MB (2008) Factors affecting the occurrence and demographics of common dolphins (*Delphinus* sp.) in the Hauraki Gulf, New Zealand. Aquatic Mammals 34: 200–211. 10.1578/AM.34.2.2008.200

[77] Parsons ECM, Fortuna CM, Ritter F, Rose NA, Simmonds MP, et al (2006) Glossary of whalewatching terms. Journal of Cetacean Research and Management 8 (Suppl): 249–251.

[78] Christiansen F, Rasmussen MH, Lusseau D (2013) Inferring activity budgets in wild animals to estimate the consequences of disturbances. Behavioral Ecology 24: 1415–1425. 10.1093/beheco/art086

[79] Dans SL, Degrati M, Pedraza SN, Crespo EA (2012) Effects of tour boats on dolphin activity examined with sensitivity analysis of Markov chains. Conservation Biology 26: 708–716. 10.1111/j.1523-1739.2012.01844.x 22624561

[80] Caswell H (2001) Matrix Population Models. New York, NY, USA: Sinaeur Associates 722 p.

[81] Fleiss JL, Levin B, Paik MC (2003) Statistical methods for rates and proportions. Balding DJ, Cressie NAC, Fisher NI, Johnstone IM, Kadane JB et al, editors. New York: Wiley Series in Probability and Statistics 792 p.

[82] R Core Team (2013) R: A Language and Environment for Statistical Computing. Vienna, Austria: R Foundation for Statistical Computing Available: http://www.R-project.org/. Accessed 2014 Nov 4.

[83] Parsons ECM, Draheim M (2009) A reason not to support whaling—A tourism impact case study from the Dominican Republic. Current Issues in Tourism 12: 397–403. 10.1080/13683500902730460

[84] Chen CL (2011) From catching to watching: Moving towards quality assurance of whale/dolphin watching tourism in Taiwan. Marine Policy 35: 10–17. 10.1016/j.marpol.2010.07.002

[85] Hughes P (2001) Animals, values and tourism—Structural shifts in UK dolphin tourism provision. Tourism Management 22: 321–329. 10.1016/S0261-5177(00)00070-4

[86] Luksenburg JA, Parsons ECM (2014) Attitudes towards marine mammal conservation issues before the introduction of whale-watching: A case study in Aruba (southern Caribbean). Aquatic Conservation-Marine and Freshwater Ecosystems 24: 135–146. 10.1002/aqc.2348

[87] Draheim M, Bonnelly I, Bloom T, Rose N, Parsons ECM (2010) Tourist attitudes towards marine mammal tourism: An example from the Dominican Republic. Tourism in Marine Environments 6: 175–183. 10.3727/154427310X12764412619046

[88] Neumann DR, Orams M (2003) Feeding behaviour of short-beaked common dolphins, *Delphinus delphis*, in New Zealand. Aquatic Mammals 29: 137–149. 10.1578/016754203101023997

[89] Neumann DR (2001) The behaviour and ecology of short-beaked common dolphins (*Delphinus delphis*) along the east coast of Coromandel Peninsula, North Island, New Zealand—With a note on their interactions with humans. PhD Thesis, Massey University 332 p.

[90] Burgess L (2006) Foraging ecology of common dolphins (*Delphinus* sp.) in the Hauraki Gulf, New Zealand. Master Thesis, Massey University 143 p.

[91] de la Brosse N (2010) Dynamics of mother-offspring common dolphin (*Delphinus* sp.) engaged in foraging activities in the Hauraki Gulf, New Zealand. Master Thesis, Massey University 94 p.

[92] Scarpaci C, Bigger SW, Corkeron PJ, Nugegoda D (2000) Bottlenose dolphins (*Tursiops truncatus*) increase whistling in the presence of ‘swim-with-dolphin’ tour operations. Journal of Cetacean Research and Management 2: 183–185.

[93] Jensen FH, Bejder L, Wahlberg M, Soto NA, Johnson M, et al (2009) Vessel noise effects on delphinid communication. Marine Ecology Progress Series 395: 161–175. 10.3354/meps08204

[94] Schaffar-Delaney A (2004) Female reproductive strategies and mother-calf relationships of common dolphins (*Delphinus delphis*) in the Hauraki Gulf, New Zealand. Master Thesis, Massey University 221 p.

[95] Young DD, Cockcroft VG (1995) Stomach contents of stranded common dolphins *Delphinus delphis* from the south-east of southern Africa. Zeitschrift Für Saugetierkunde-International Journal of Mammalian Biology 60: 343–351.

[96] Meynier L, Pusineri C, Spitz J, Santos MB, Pierce GJ, et al (2008) Intraspecific dietary variation in the short-beaked common dolphin *Delphinus delphis* in the Bay of Biscay: Importance of fat fish. Marine Ecology Progress Series 354: 277–287. 10.3354/meps07246

[97] Young DD, Cockcroft VG (1994) Diet of common dolphins (*Delphinus delphis*) off the south-east coast of southern Africa: Opportunism or specialization? Journal of Zoology 234: 41–53. 10.1111/j.1469-7998.1994.tb06055.x

[98] Lusseau D (2003) Male and female bottlenose dolphins *Tursiops* spp. have different strategies to avoid interactions with tour boats in Doubtful Sound, New Zealand. Marine Ecology Progress Series 257: 267–274.

[99] Dwyer SL, Visser IN, Tezanos-Pinto G, Meissner AM, Berghan J, et al (2014) Overlooking a potential hotspot for a nationally endangered dolphin: A consequence of user-pays research. Endangered Species Research 25: 97–114. 10.3354/esr00613

[100] Zaeschmar JR, Visser IN, Fertl D, Dwyer SL, Meissner AM, et al (2014) Occurrence of false killer whales (*Pseudorca crassidens*) and their association with common bottlenose dolphins (*Tursiops truncatus*) off northeastern New Zealand. Marine Mammal Science 30: 594–608. 10.1111/mms.12065

[101] Neumann DR, Leitenberger A, Orams MB (2002) Photo-identification of short-beaked common dolphins (*Delphinus delphis*) in north-east New Zealand: A photo-catalogue of recognisable individuals. New Zealand Journal of Marine and Freshwater Research 36: 593–604. 10.1080/00288330.2002.9517115

[102] Rankmore K (2015) Abundance, site fidelity and social structure of common dolphins (*Delphinus* sp.) in the Hauraki Gulf, New Zealand. PhD Thesis, Massey University (In prep.).

[103] Constantine RL (1995) Monitoring the commercial swim-with-dolphin operations with the bottlenose (*Tursiops truncatus*) and common dolphins (*Delphinus delphis*) in the Bay of Islands, New Zealand. Master Thesis, University of Auckland 105 p.

[104] Barr K (1997) The impacts of marine tourism on the behaviour and movement patterns of dusky dolphins (*Lagenorhynchus obscurus*), at Kaikoura, New Zealand. Master Thesis, University of Otago 97 p.

[105] Bejder L, Dawson SM, Harraway JA (1999) Responses by Hector’s dolphins to boats and swimmers in Porpoise Bay, New Zealand. Marine Mammal Science 15: 738–750. 10.1111/j.1748-7692.1999.tb00840.x

[106] Markowitz TM, DuFresne S, Würsig B (2009) Tourism effects on dusky dolphins at Kaikoura, New Zealand. Wellington, New Zealand: Department of Conservation 93 p.

[107] Lusseau D (2006) The short-term behavioral reactions of bottlenose dolphins to interactions with boats in Doubtful Sound, New Zealand. Marine Mammal Science 22: 802–818. 10.1111/j.1748-7692.2006.00052.x

[108] Nowacek SM, Wells RS, Solow AR (2001) Short-term effects of boat traffic on bottlenose dolphins, *Tursiops truncatus*, in Sarasota Bay, Florida. Marine Mammal Science 17: 673–688. 10.1111/j.1748-7692.2001.tb01292.x

[109] Miller LJ, Solangi M, Kuczaj SA (2008) Immediate response of Atlantic bottlenose dolphins to high-speed personal watercraft in the Mississippi Sound. Journal of the Marine Biological Association of the United Kingdom 88: 1139–1143. 10.1017/S0025315408000908

[110] Wells RS, Scott MD (1997) Seasonal incidence of boat strikes on bottlenose dolphins near Sarasota, Florida. Marine Mammal Science 13: 475–480. 10.1111/j.1748-7692.1997.tb00654.x

[111] Nichols C, Stone G, Hutt A, Brown J, Yoshinaga A (2001) Observations of interactions between Hector’s dolphins (*Cephalorhynchus hectori*), boats and people at Akaroa Harbour, New Zealand. Wellington, New Zealand: Department of Conservation 49 p.

[112] Thompson PM, Wilson B, Grellier K, Hammond PS (2000) Combining power analysis and population viability analysis to compare traditional and precautionary approaches to conservation of coastal cetaceans. Conservation Biology 14: 1253–1263. 10.1046/j.1523-1739.2000.00099-410.x

[113] Wilson B, Hammond PS, Thompson PM (1999) Estimating size and assessing trends in a coastal bottlenose dolphin population. Ecological Applications 9: 288–300. 10.1890/1051-0761(1999)009[0288:ESAATI]2.0.CO;2

